# 2363: Ventriculo-arterial coupling and left ventricular mechanical work in systolic and diastolic heart failure

**DOI:** 10.1017/cts.2017.133

**Published:** 2018-05-10

**Authors:** Leo Buckley, Justin Canada, Salvatore Carbone, Cory Trankle, Michele Mattia Viscusi, Jessica Regan, Dave Dixon, Nayef Abouzaki, Sanah Christopher, Hayley Billingsley, Dinesh Kadariya, Ross Arena, Antonio Abbate, Benjamin Van Tassell

**Affiliations:** Virginia Commonwealth University, Richmond, VA, USA

## Abstract

OBJECTIVES/SPECIFIC AIMS: Our goal was to compare the ventriculo-arterial coupling and left ventricular mechanical work of patients with systolic and diastolic heart failure (SHF and DHF). METHODS/STUDY POPULATION: Patients with New York Heart Association Functional Class II-III HF symptoms were included. SHF was defined as left ventricular (LV) ejection fraction<50% and DHF as >50%. Analysis of the fingertip arterial blood pressure tracing captured with a finger plethysmography cuff according to device-specific algorithms provided brachial artery blood pressure and stroke volume. LV end-systolic volume was measured separately via transthoracic echocardiography. Arterial elastance (Ea), a measure of pulsatile and nonpulsatile LV afterload, was calculated as LV end-systolic pressure (ESP)/end-diastolic volume. End-systolic elastance (Ees), a measure of load-independent LV contractility, was calculated as LV ESP/end-systolic volume. Ventriculo-arterial coupling (VAC) ratio was defined as Ea/Ees. Stroke work (SWI) was calculated as stroke volume index×LV end-systolic pressure×0.0136 and potential energy index (PEI) as 1/2×(LV end-systolic volume×LV end-systolic pressure×0.0136). Total work index (TWI) was the sum of SWI+PEI. RESULTS/ANTICIPATED RESULTS: Patients with SHF (n=52) and DHF (n=29) were evaluated. Median (IQR) age was 57 (51–64) years. There were 48 (58%) and 59 (71%) patients were male and African American, respectively. Cardiac index was 2.8 (2.2–3.2) L/minute and 3.0 (2.8–3.3) L/minute in SHF and DHF, respectively (*p*=0.12). Self-reported activity levels (Duke Activity Status Index, *p*=0.48) and heart failure symptoms (Minnesota Living with Heart Failure Questionnaire, *p*=0.55) were not different between SHF and DHF. Ea was significantly lower in DHF compared with SHF patients [1.3 (1.2–1.6) vs. 1.7 (1.4–2.0) mmHg; *p*<0.001] whereas Ees was higher in DHF vs. SHF [2.8 (2.1–3.1) vs. 0.9 (0.7-1.3) mmHg; *p*<0.001). VAC was 1.8 (1.3–2.8) in SHF Versus 0.5 (0.4–0.7) in DHF (*p*<0.001). Compared with SHF, DHF patients had higher SWI [71 (57–83) vs. 48 (39–68) gm×m; *p*<0.001) and lower PEI [19 (12–26) vs. 44 (36–57) gm×m; *p*<0.001]. TWI did not differ between SHF and DHF (*p*=0.14). Work efficiency was higher in DHF than SHF [0.80 (0.74–0.84) vs. 0.53 (0.46–0.64); *p*<0.001]. DISCUSSION/SIGNIFICANCE OF IMPACT: The results underscore the differences in pathophysiology between SHF and DHF patients with similar symptom burden and exercise capacity. These results highlight the difference in myocardial energy utilization between SHF and DHF.

